# Emergency Evacuation Plan for Hazardous Chemicals Leakage Accidents Using GIS-based Risk Analysis Techniques in South Korea

**DOI:** 10.3390/ijerph16111948

**Published:** 2019-06-01

**Authors:** Byungtae Yoo, Sang D. Choi

**Affiliations:** 1 Accident Prevention and Assessment Division, National Institute of Chemical Safety, 90 Gajeongbuk-ro, Yuseong-gu, Daejeon 305-343, Korea; flyduck@korea.kr; 2 Department of Occupational & Environmental Safety & Health, University of Wisconsin-Whitewater, Whitewater, WI 53190, USA

**Keywords:** chemical accident, emergency response, evacuation plan, geographic information system, real-time risk assessment, decision making, consequence analysis

## Abstract

Despite improvements in chemical safety management systems, incidents involving the release of hazardous chemicals continue to happen. In some cases, they result in the evacuation of residents. For hazardous chemical release accidents, an evacuation plan needs to be selective enough to consider both the indoor and outdoor concentrations of nearby buildings and the time in which the maximum allowable concentration may occur. In this study, a real-time risk analysis tool was developed based on the geographic information system (GIS) in order to establish the emergency response and risk communication plan for effectively assisting decision-making personnel. A selective evacuation plan was also established by a proposed assessment module considering the indoor/outdoor pollution concentration of buildings and the release duration time of chlorine gas leakage. The GIS-based simulated modules were performed based on eleven buildings of Ulsan city, located near an industrial cluster and home to a high population density. As a result of the simulated real-time risk assessment, only four buildings were affected by chlorine gas concentration according to wind direction and diffusion time. In addition, it was considered effective to establish an indoor/outdoor evacuation plan as opposed to an outdoor evacuation plan which is outside the range of the damage. Subsequently, an emergency evacuation plan was established with the concentration of a hazardous chemical according to the decision-making matrix. This study can enlighten the real-time emergency risk assessment based on GIS while effectively supporting the emergency action plans in response to the release of hazardous chemicals in clustered plants and the community.

## 1. Introduction

Many of the hazardous chemical substances used by industries are potentially dangerous if accidentally released into the atmosphere because they can be either toxic or flammable, or both [1]. The amount of hazardous chemical used in South Korea is increasing (with a 39% average annual increase) due to development in high-tech industries such as semiconductors and electronic devices [2]. Hazardous chemicals such as hydrofluoric acid, nitric acid, and chlorine gas are widely used in the South Korean industry for manufacturing the electronic devices during the chemical treatment such as etching and anodizing [3,4]. In the United States, the Occupational Safety and Health Administration (OSHA), Environmental Protection Agency (EPA), Federal Emergency Management Agency (FEMA), and Chemical Safety and Hazard Investigation Board (CSB) restricted the usage and production of toxic chemicals without permission and required careful handling according to the safety regulations [5,6,7].

However, a highly hazardous chemical, such as hydrofluoric acid, release accident for instance unfortunately occurred in the South Korean city of Gumi in 2012 [8,9]. After the chemical leak accident in the city of Gumi, hydrogen fluoride continued to spread from the worksites to the local occupants. Due to the inadequate disaster planning and emergency evacuation management system, more than 3600 residents sought medical treatment for rashes, nausea, chest pain, and sore eyes, and over 300 residents had to be displaced to nearby temporary shelters. Crops and livestock near the site were also affected. The damage was estimated to be more than 17.7 billion KRW (20 million USD), and 323.8 ha of crops were destroyed [4,10,11]. Chemical plants were located in clusters due to the environmental conditions, safety requirements and related regulations [12,13]. These enormous damages were caused by the concentrated South Korean industrial structure (multi-plant cluster) and high population density. It should be noted that emergency planning and response management can play a key role in reducing risk by avoiding fatalities and injuries, as well as protecting the environment [6]. According to OSHA 1910.38 (Emergency Action Plans) and Appendix to Subpart E (Exit Routes and Emergency Plan), at the time of an emergency, employees should know what type of evacuation is necessary and what their role is in carrying out the emergency evacuation plan [14,15]. 

When establishing emergency action plans for chemical plants, it is crucial to be able to predict the dispersal of hazardous chemical substances. The adequate prediction of hazardous chemical distributions can also facilitate evacuations and victim rescue in affected areas. Whether to “recommend evacuation” or “shelter-in-place” is one of the most critical decisions facing decision-makers (e.g., emergency managers) as they respond to a hazardous chemical release incident [1,16,17]. To create an appropriate emergency evacuation procedures (e.g., exit routes, critical plant operations, rescue or medical duties) that could minimize casualties in a hazardous chemical release, the emergency response plan needs to consider indoor/outdoor pollution concentrations and the time-to-maximum allowable concentrations of nearby buildings, based on release duration. However, many factory plants in South Korea have prepared an overly simplistic emergency plan that merely evacuates people from the potential damage range. Furthermore, the industrial structure is based on clusters often including in excess of hundreds of factories. Thus, if chemical accidents have occurred, successive damage presents a concern. Further, residential districts are often close to industrial clusters, within approximately 5 km. Because of the local characteristics of South Korea, the damage to people in the residential area near the factory occurred not only outside the building but also inside the evacuated building [18].

With above local problems in South Korea, major accidents with cross-plant consequences (crisis situations) require the involvement of emergency response management (incident commanders, technical safety experts, fire brigades, etc.) from different plants, thus the organization and implementation of such emergency planning needs a different emergency response strategy for major accidents. In chemical industrial areas, considerably less attention has been given to multi-plant emergency response planning. In these industrial areas, other plants and nearby communities may be affected in addition to the company where the major accident takes place. 

A close cooperation system is needed to prevent and respond to chemical accidents for regions with a high concentration of petrochemical factories [19,20]. Particularly, toxic gases may be released and spread to nearby workspaces, and in some cases, the spread of toxic gases is fast and evacuating to faraway places is impossible. Therefore, it is necessary to develop systems and evaluation tools in order to establish appropriate emergency response strategies, such as setting up indoor evacuation systems in consideration of the ventilation rate of building interiors and sharing accident scenarios in advance.

Risk, the product of likelihood (or frequency) and consequence (or severity) of an event, can be defined broadly as a condition in which there is a possibility that persons or property could experience adverse consequences [21]. Risk analysts have a responsibility to convey their assessment to decision makers (e.g., emergency managers, fire brigades, emergency management service (EMS) responders, etc.) who determine what action to take in response to the risk that the analyst has characterized. Risk communication should be a process in which stakeholders share information about hazards affecting a community. The use of the term sharing is important because risk analysts and emergency managers must understand how different segments of the population at risk think about a hazard if they are to be effective in communicating with their audience. These population segments include businesses and households that are vulnerable to a specific hazard, as well as community and industry personnel who are responsible for managing a hazard in ways that reduce the risk to a level that is acceptable to the community [22]. Individual risk perception is consequently and most likely affected by errors and it is also rather far from the effective risk level. It is important to emergency planning and disaster analysis to help an emergency response planner be aware of possible biases and ensuring that decisions he/she makes are rational and discreet [23,24]. 

The emergency evacuation plan plays a key role in disaster management and successful evacuation [25]. In particular, chemical factories require effective emergency response strategies in order to minimize damage to workers and local residents [25,26,27,28]. To establish successful indoor and outdoor evacuation strategies, weather conditions and changes in toxic gas concentrations must be evaluated by considering the ventilation rates of neighboring buildings. However, many previous studies have focused on the estimation of evacuation routing optimization or emergency response procedures from natural disasters or man-made disasters [29,30,31,32,33,34]. Only few studies proposed an emergency response decision making tool in order to prioritize credible accident scenarios, identify emergency levels, and determine emergency action plans accordingly [6]. 

The purpose of this study was to develop a real-time emergency risk assessment and consequently to describe the logical and practical emergency response plan with risk analysis of indoor/outdoor toxic concentration and risk communication plan for effectively assisting the decision-making personnel. An assessment module was proposed based on the geographic information system (GIS)-based risk analysis tool, including the indoor and outdoor simulated modules to perform the risk analysis under high population density and to predict indoor concentration of toxic gas with nearby building. Ulsan in South Korea was selected as a specimen for hazard analysis due to its industrial cluster and high population density. In addition, an emergency evacuation plan was established with a simulated concentration of a hazardous chemical according to the decision-making matrix.

## 2. Materials and Methods

### 2.1. Outdoor/Indoor Concentration Analysis 

To establish an evacuation plan for the release of hazardous chemicals from containers, the atmospheric diffusion of leaked substances needs to be assessed in order to predict indoor and outdoor concentrations at nearby buildings. The existing diffusion models mostly use a Gaussian approach and thus can only assess the distribution of concentrations assuming a steady state. They cannot consider the complex topography around chemical plants. Computational fluid dynamics (CFD)-based diffusion models, which were used to improve upon Gaussian models, required too much processing time to make an assessment [35,36,37,38]. Accordingly, CFD-based models could not be effectively used to establish evacuation plans that demanded real-time diffusion assessments. 

To improve these limitations, this study adopted a Lagrangian diffusion model that enabled real-time assessment, as well as incorporated nearby terrain and temporal chemical concentration variations. This model was used to predict the indoor concentrations of toxic gas within nearby buildings. 

In this study, the potential dispersion region is divided into 100 m grids and the spread of chemical substances on wind fields is simulated, assuming that the leaking chemical substances are particulates. The unit of particulate dispersion is processed by dividing each wind field to 5~10 units; as such, the particulate dispersion unit is made up of 10~20 m units. As a result, the performance of this particle analysis method is faster than that of the existing CFD method. This is a very important characteristic for dispersion plans which require real-time information. The newly proposed assessment module includes duration time, air changes per hour (ACH) and nearby buildings. The indoor and outdoor concentrations (ppm) could be obtained during when a toxic gas initially reaches a particular building and then escapes the area. However, the tool is designed to manually input the ventilation rate of buildings under the condition that the user already knows the ventilation rate of the building. 

The toxic gas arrival time, weather conditions and building ventilation rate cause changes in the indoor pollutant concentration; as such, to assess whether buildings can be used as places for evacuation, the tool must be able to measure the pollutant concentration by considering these items.

#### 2.1.1. Outdoor Concentration

The mass flow rate of toxic gas at the leak point is calculated by [39]

(1) $${\left(Q_{m}\right)}_{choked}=\;C_{0}AP_{0}\;\sqrt{\frac{rg_{c}M}{R_{g}T_{0}}{\left(\frac{2}{r+1}\right)}^{\frac{r+1}{r-1}}}\;\mathrm{or}\;\;\left(Q_{m}\right)=\;C_{0}AP_{0}\;\sqrt{\frac{2g_{c}M}{R_{g}T_{0}}\left(\frac{r}{r-1}\right)\left[{\left(\frac{P}{P_{0}}\right)}^{2/r}\;-{\left(\frac{P}{P_{0}}\right)}^{\left(r+1\right)/r}\right]}$$

where Q_m_ is the mass rate of flow, C_0_ is the leak drag coefficient, A is the leak area, P_0_ is the release pressure, r is the heat capacity ratio, g_c_ is the gravitational constant, M is the molecular weight, R_g_ is the ideal gas constant, T_0_ is the release temperature, and P is the ambient pressure.

A Lagrangian diffusion model was used to simulate the external diffusion of toxic gases. The model assesses the distribution of toxic gas concentration by considering time and terrain within a predicted diffusion range. Compared to frequently used Gaussian models, the Lagrangian model is much more practical. The Lagrangian model considered the following factors to calculate the effects of terrain on wind [40,41,42]:

The kinematic Effect was calculated by Equation (2);

(2) $$W=\left(V\cdot \nabla h_{t}\right)\exp\left(-kz\right)$$

where V is the domain-mean wind, h_t_ is the terrain height, k is a stability-dependent coefficient of exponential decay, and z is the vertical coordinate. The slope flow was calculated by Equation (3);

(3) $$S=S_{e}{\left[1-\exp\left(-\frac{x}{L_{e}}\right)\right]}^{1/2}\;S_{e}={\left[hg\left(\frac{\Delta \theta }{\theta }\right)sin\alpha /\left(C_{D}+k\right)\right]}^{1/2}\;L_{e}=h/\left(C_{D}+k\right)$$

where S_e_ is the equilibrium speed of the slope flow, L_e_ is an equilibrium length scale, x is the distance to the crest of the hill, Δθ is the potential temperature deficit with respect to the environment, θ is the potential temperature of the environment, C_D_ is the surface drag coefficient, h is the depth of the slope flow, α is the angle of the terrain relative to the horizontal, k is the entrainment coefficient at the top of the slope flow layer, and g is the gravitational acceleration constant (9.8 m s^−2^). The blocking effect was calculated by Equation (4);

(4) $$\mathrm{Fr}=\frac{V}{N\Delta h_{t}}\;\Delta h_{t}=({h_{\max})}_{ij}-{\left(z\right)}_{ijk}$$

where Fr is the local Froude number, V is the wind speed at the grid point, N is the Brunt-Vaisala frequency, $\Delta h_{t}$ is an effective obstacle height, $({h_{\max})}_{ij}$ is the highest gridded terrain height within a radius of influence (TERRAD) of the grid point (i, j), and ${\left(z\right)}_{ijk}$ is the height of level k of grid point (i, j) above ground.

#### 2.1.2. Indoor Concentration

Indoor concentration is described as follows [43,44]. The rate of change in the indoor concentration C(t) at t minutes after t_a_ is equal to the effective infiltration rate λ multiplied by the difference between the outdoor concentration C_0_ during cloud passage and the indoor concentration C(t),

(5) $$\frac{dC\left(t\right)}{dt}=\;\lambda \left[C_{0}-C\left(t\right)\right]$$

**Figure 1:**
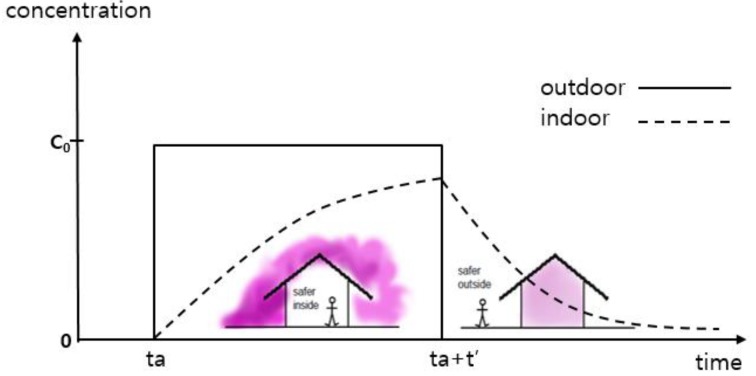
A typical time series of an indoor concentration (dashed line) for an outdoor concentration (solid line) with a top-hat [18,43].

Figure 1 shows a “top-hat” form for the progression of the indoor and outdoor concentrations over time. The concentrations can initially be higher outdoors, but later, the concentrations become higher inside. However, these relationships depend on the details of the building ventilation.

## 3. Results and Discussion

### 3.1. Scenario Selection

**Figure 2:**
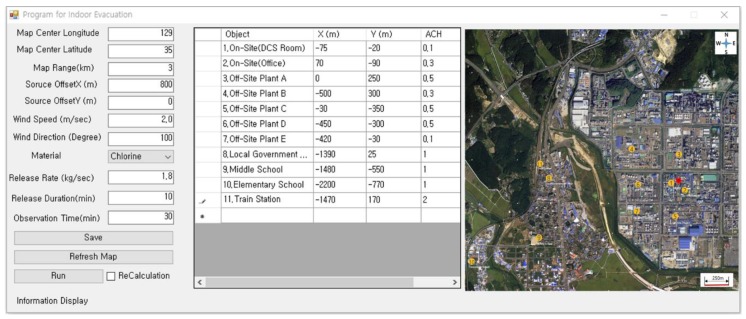
Scenario input data for the simulation.

This study assumed a chlorine gas release from the broken lower flange of a drying tower during the very dangerous drying process, while chlorine was being fabricated. The details of the release conditions are listed in Table 1. An accident scenario can be affected by accident chemicals, weather conditions, and process conditions. In this study, we assumed an accident lasting 10 minutes involving the release of chlorine, and the affected area was selected as a place including a residential area, school, and train station. In the area surrounding the release point (S), 11 buildings were selected. These buildings included two plants dealing with chlorine, five other plants, one local community center, one elementary school, one middle school, and one train station, as shown in Table 2. The ventilation rate was set to four levels ranging from 0.1 to 1.0. Since the train station had a lot of visitors, its ventilation ranged from 0.5 to 2.0, as shown in Figure 2. 

### 3.2. Diffusion Analysis of Chlorine Gas via Developed Module

**Figure 3:**
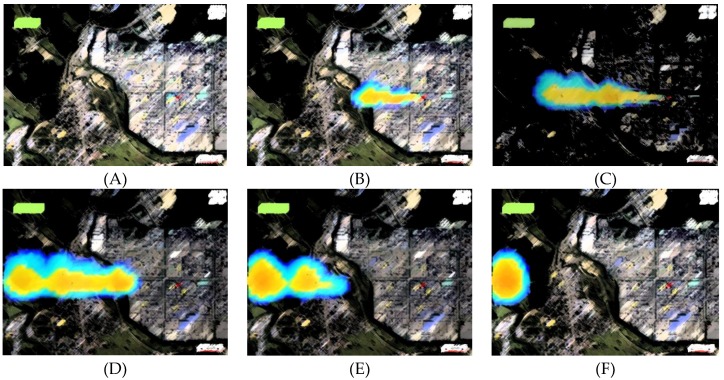
Dispersion results relative to time ((**A**) 1 min, (**B**) 5 min, (**C**) 10 min, (**D**) 15 min, (**E**) 20 min, and (**F**) 30 min)).

The assessment results of chlorine gas diffusion, based on release duration (1 min, 5 min, 15 min, 20 min, and 30 min) show that four (①, ⑦, ⑧, ⑪) out of eleven buildings fell within the diffusion range of chlorine gas, as shown in Figure 3 and Table 3. First, on-site (DCS Room, ①) could be classified as indoor because it is below the allowable standard. Second, off-Site (Plant E, ⑦), where the chlorine gas reached only 13.8 seconds (ACH 0.1), it was necessary to stay indoors for a time that made it difficult to move to an outside shelter, and response measures or prompt rescue requests were required. Lastly, the local government office(⑧) and train station(⑪), which are 1390 m and 1470 m away from the leakage point, show the initial arrival time of chlorine gas to 10.2 minutes (ACH 0.3) and 13.2 minutes (ACH 1.0) respectively. Therefore, escaping from the area was safer than staying in the building.

#### 3.2.1. On-Site (①)

**Figure 4:**
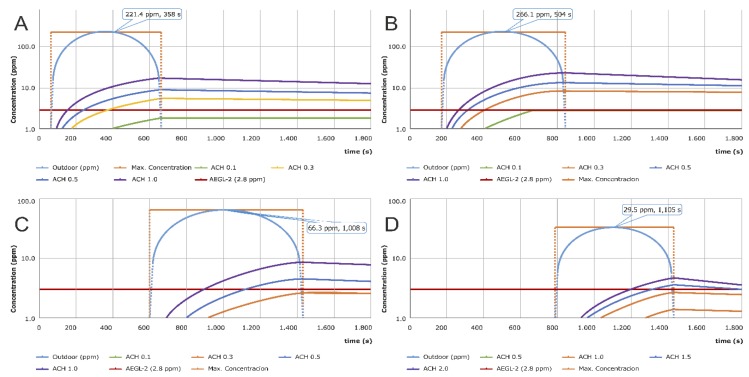
Concentrations at various ACHs (**A**) On-site, (**B**) Off-site (Plant E), (**C**) Local government office, and (**D**) Train station.

In outdoor locations, the chlorine gas arrived at 61 s, the highest concentration of 221.4 ppm was observed at 358 s, and the gas completely dissipated at 656 s. In indoor locations, the initial inflow of chlorine gas was between 62 (ACH 1.0) and 77 s (ACH 0.1). When the ventilation rate was 0.1, the chlorine gas concentration was below the allowable level (2.8 ppm). On the other hand, when the ventilation rate was 1.0, the chlorine concentration rose as high as 16.8 ppm at 660 s. For the case of the indoor environment, ACH 0.1 had a maximum concentration of 1.8 ppm (642 s), which was below allowable levels. This indicated that indoor evacuation could be an effective emergency response. However, when the ventilation rates were 0.3, 0.5, and 1.0, the outdoor pollution concentrations exceeded the allowable level within a short time, making outdoor evacuation impossible. In this case, a secondary rescue or additional measure was required after the primary indoor evacuation, as shown in Figure 4A. 

#### 3.2.2. Off-Site Plant E (⑦)

The highest outdoor pollution concentration of Plant E was 266.1 ppm, achieved at 504 s. The initial reach time of chlorine gas was 180 s, the allowable concentration was 182 s, and the chlorine gas dissipated by 840 s. Inside the building, the initial inflow time ranged from 182 (ACH 1.0) to 192 s (ACH 0.1). When the ventilation rate was 0.1, the chlorine gas concentration was below the allowable level. On the other hand, when the ventilation rates were 0.3, 0.5, and 1.0, the allowable concentration was reached at 397, 311, and 246 s, respectively. Moreover, the outdoor concentration reached the allowable level in only 182 s and rose to 266.1 ppm at 504 s. As the chlorine gas remained in the area for 660 s, outdoor evacuation was not possible. Accordingly, after primary indoor evacuation, a secondary rescue or additional measure should be taken by the initial response agency, as shown in Figure 4.

#### 3.2.3. Local Government Office (⑧)

The initial reach time of chlorine gas was 601 s, and the inflow into indoor space occurred between 605 (ACH 1.0) and 650 s (ACH 0.1). When the ventilation rates were 1.0 and 0.3, the highest pollution concentrations were 0.8 and 2.5 ppm, respectively, both of which were below the allowable concentration. Accordingly, the indoor evacuation was sufficient for emergency response. Since the initial reach time exceeded 10 min, outdoor evacuation could be an alternative, if necessary, as shown in Figure 4C.

#### 3.2.4. Train Station (⑪)

The chlorine gas initially arrived at the station in 781 s and passed the location completely in 1439 s. The initial inflow into the indoor space occurred between 787 (ACH 2.0) and 805 s (ACH 0.5). When ventilation rates were 0.5 and 1.0, the concentration was below the allowable level. Accordingly, indoor evacuation could be an effective response measure. However, since the initial reach time exceeded 10 min and the station was typically busy, the inflow of chlorine gas could not be effectively controlled. Consequently, the outdoor evacuation seemed to be more effective, as shown in Figure 4D.

### 3.3. Determination of Emergency Levels

**Figure 5:**
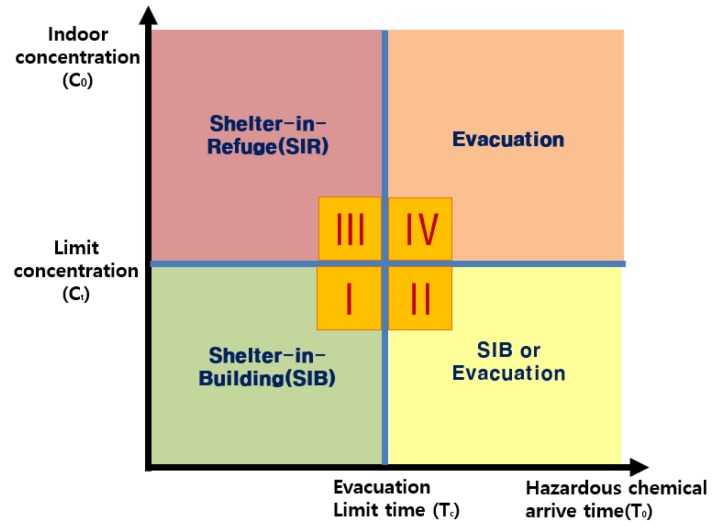
Evacuation matrix [40].

A decision matrix was proposed that can estimate the range of damage caused by the leakage of hazardous chemicals and establish a selective evacuation plan depending on the evacuation behavior procedure. The decision making for the selective evacuation plan was established by considering the following parameters in a comprehensive manner; release time, indoor and outdoor concentration, downwind distance, air changes per hour (ACH. Therefore, the emergency evacuation plan was classified into (1) shelter in place, (2) shelter in refuge, and (3) evacuation, as shown in Figure 5. In addition, decision supporting matrix was classified into four stage (I~IV). The X axis was set as a time parameter and classified into hazardous chemical arrival time (T_0_) and evacuation limit time (T_c_). The Y axis is classified into indoor pollution concentration (C_0_) and limit concentration (C_t_). 

This matrix can be used when:

I The toxic gas diffusion time is fast and the evacuation time is insufficient
II The pollutant concentration is below the allowable concentration level, or the evacuation time is sufficient
III Rescue activities must be performed simultaneously because the evacuation time is insufficient and as the concentration is above the allowable level
IV Outdoor evacuation is possible because the location is sufficiently far from the accident site or the damage diffusion speed is not fast.

As shown in the chlorine gas release case results, four out of the eleven areas of interest were included in the scope of damage impact. In order for the evacuation plans of the four locations to be effective, these plans need to be different to the conventional uniform outdoor evacuation. Further, differentiated evacuation plans need to be implemented, such as indoor evacuation, indoor evacuation followed by outdoor evacuation, and outdoor evacuation. For the other seven locations, the primary response should be indoor evacuation followed by outdoor evacuation plan when necessary in consideration of the accident progress. Effective evacuation plans can be created by considering workspace conditions, such as wind direction, wind speed, accident material and indoor pollution concentration level by the ventilation rate of buildings in the area of interest.

### 3.4. Emergency Response and Evacuation Action Plan

Based on the newly developed module, a diffusion analysis of chlorine gas was performed on wind direction and diffusion time. In addition, an emergency evacuation plan was presented in a high-density population area based on the computed result. As a result of real-time modeling considering the wind direction, only four out of eleven buildings located in the direction of the damage affected area were included in the damage scope. In the case of four buildings around the damage affected area, it was considered effective to establish an indoor and outdoor evacuation plan other than the outdoor evacuation plan which is outside the range of the existing damage represented by the circle on the Korea Off-site Risk Assessment Supporting Tool (*KORA*) system [45]. In addition, residents around seven other buildings were found to be able to prevent damage only by indoor evacuation. Each emergency level indicates the planning requirements and the people responsible when a specific accident scenario occurs within the chemical cluster.−Since the emergency response strategies are defined for the different types of emergency situations, scenario specific detailed action plans are established. These action plans are part of a pre-plan technique that is widely used in the chemical industry [46]. The action plans are presented in the form of a table that shows a series of steps (based on the emergency strategies) that need to be followed when responding to an emergency situation based on its dispersion map (impact zones) and the evaluated emergency levels at individual plants within the industrial park. The table also includes the necessary resources (internal and external) such as the emergency communication systems, the alarm system and the emergency equipment (personal protective clothing, first aid, firefighting systems and etc.), as well as the responsible personnel (on-site and off-site) assigned to fulfill the responsive functions.

Since the emergency levels for each chemical company are identified, the response strategies can also be established and scenario specific detailed action plans can be set for the selected scenarios. Table 4 and Table 5 report the role of on-site and offsite responders in managing the emergency situation and the needed resources.

Practical approaches to risk management should build on both the technical know-how of professionally trained people and the knowledge and perceptions of those at risk. Instituting risk management plans that ignore local knowledge, local political structures and local priorities will not be effective; neither will plans built largely on local knowledge without external technical input where relevant. A compromise is needed. This means formulating risk management plans into which the community has input, and which local people can help to implement. Consultation, discussion and negotiation are vital aspects of such approaches. In emergency situations, prompt emergency response and evacuation actions are essential to save human lives. Long-term planning with community participation in order to make people less vulnerable in emergency situations is also imperative. Risk management is a two-way process that must take into account both “the hazard and its impact on people” and “the people’s responses to the situation” [47]. 

To continuously improve the emergency response system against hazardous chemical accidents, the role of on-site coordinators is critical for assessing indoor and outdoor pollution concentrations of buildings by considering the release duration of toxic gases. For instance, a ‘Chemical Accident Investigation Committee’ should be established at a national level, and positive industry-university-research centered cooperation and a culture of safety should also be formed. Control is not achieved by an individual person but instead requires good organization, which should be both consistent and flexible. Additionally, the on-site coordinator needs to have knowledge about relevant fields so that he or she can handle the accident from an overall perspective by assessing and monitoring the accident after the initial response. Furthermore, the on-site coordinator should function as a technological supporter by providing professional advice related to containing the accident and implementing remediation measures after the accident, including limiting pollution and removing hazardous chemicals [4]. 

## 4. Conclusions

This study conducted a simulated emergency risk analysis and consequently described logical and practical emergency action plans for effectively assisting the decision-making personnel in response to a hazardous chemical leakage accident. We revealed a real-time damage assessment module based on the geographic information system (GIS) considering toxic chemical leakage scenarios and classified the selective emergency evacuation plans by predicting the time to reach the allowable concentration in the surrounding buildings. According to the simulated chlorine gas leakage risk assessment in clustered chemical plants, only four out of eleven buildings around the accident area were included in the damage scope. In the four sites included in the scope of influence, the indoor pollution concentration value of the on-site closest to the leakage point could be evacuated to the indoor only below the allowable standard. In the off-site (e.g., Plant E), the chlorine gas reached only 13.8 seconds, so it was necessary to stay indoor for a time that was difficult to move to an outside shelter, and response measures or prompt rescue requests were required. The local government office and train station, which are 1390 m and 1470 m apart from the leakage point, analyzed the initial arrival time of chlorine gas to 10.2 minutes and 13.2 minutes respectively, whereas escaping from the area was safer than stay in the building. This study demonstrated the determination of emergency levels computed by the GIS based visual simulation, subsequently emergency evacuation action classifications (shelter in place, shelter in refuge, evacuation), and on/off-site emergency evacuation alarm systems. This study presents a new approach for an emergency evacuation plan with risk assessment that can predict the allowable toxic concentration in neighboring areas or buildings. Its results are of high interest for further development, scientific and effective emergency management planning for workers and the public in chemical plant parks, in particular when considering new and emerging multiple risks, which will become a future research direction. All in all, although this study’s limitation of a hazardous chemical diffusion with only computer simulation modeling, the findings of the current study can enlighten the prospects of real-time risk assessment, possibly supporting the decision makers’ emergency response and timely evacuation actions in response to the release of hazardous chemicals in clustered plants and the community. 

## Figures and Tables

**Table 1:** Release conditions for chemical leakage accident scenario.

Parameter	Data
Hazardous chemical	Chlorine (Cl_2_)
Release rate	1.8 kg s^−1^
Release duration	10 min
Observation time	10 min
Wind speed	2 m s^−1^
Wind direction	100°
Acute Exposure Guideline Levels-2 (AEGL-2)	2.8 ppm (10 min)

**Table 2:** Building locations relative to toxic gas release point.

Building		Distance from Source (m)		ACH	
		X	Y	Min	Max
①	On-Site	−75	−20	0.1	1.0
②	On-Site (Office)	70	−90	0.1	1.0
③	Off-Site Plant A	0	250	0.1	1.0
④	Off-Site Plant B	−500	300	0.1	1.0
⑤	Off-Site Plant C	−30	−350	0.1	1.0
⑥	Off-Site Plant D	−450	−300	0.1	1.0
⑦	Off-Site Plant E	−420	−30	0.1	1.0
⑧	Local Government Office	−1390	25	0.1	1.0
⑨	Middle School	−1480	−550	0.1	1.0
⑩	Elementary School	−2200	−770	0.1	1.0
⑪	Train Station	−1470	170	0.5	2.0

**Table 3:** Indoor and outdoor concentrations of each affected building.

Division	Indoor																Outdoor			
ACH	0.1			0.3			0.5				1.0				1.5	2.0				
Building	①	⑦	⑧	①	⑦	⑧	①	⑦	⑧	⑪	①	⑦	⑧	⑪	⑪	⑪	①	⑦	⑧	⑪
First reach time	77	192	650	66	184	617	64	183	610	805	62	182	605	793	789	787	61	180	601	781
2.8 ppm (AEGL-2)	-	-	-	366	397	-	244	311	1162	-	152	246	881	-	1285	1159	62	182	609	797
Max ppm/time (s)	1.8/642	2.8/831	0.8/1345	5.3/652	8.2/835	2.5/1431	8.7/654	13.4/835	4.0/1422	1.3/1429	16.8/660	25.7/839	7.6/1431	2.4/1416	3.5/1432	4.5/1440	221.4/358	266.1/504	66.3/1008	29.5/1105
Dissipated	-		-	-		-	-		-	-	-	-	-		-	-	659	840	1439	1439

**Table 4:** Emergency action.

Process	Task	Task
Detect and report of leakage	Connect control room, Gas alarm check Emergency communication system operation	Worker
Leakage prevention	Operating adsorption tower, Wear protective equipment, Operating containments	Worker Utility manager
Preventive measures against spread	Prevention vapor diffusion by water spray Prevention of secondary spread by utilizing sandbag	Worker, EHS Team
Recovery action	Transfer pollutants to wastewater treatment system Carry out pollutants by waste transportation car	Worker, EHS Team

**Table 5:** Emergency alarm system.

Emergency Type	Emergency Alarm	Description
Alert	3-minute-long sound	In case of process abnormal or unsafe situation
Gas Release	Repetition long and short sound	In case of leakage flammable or toxic gas
Fire	5-second-long sound × 3 times	In case of fire
Evacuation	3 short sound continuity	In case of possible to catastrophic personal injury by explosion or toxic gas leakage
Clear	1-minute-long sound	Release status
